# Nutritional Component Analyses in Different Varieties of *Actinidia eriantha* Kiwifruit by Transcriptomic and Metabolomic Approaches

**DOI:** 10.3390/ijms231810217

**Published:** 2022-09-06

**Authors:** Huimin Jia, Junjie Tao, Wenqi Zhong, Xudong Jiao, Shuangshuang Chen, Mengting Wu, Zhongshan Gao, Chunhui Huang

**Affiliations:** 1 College of Agronomy, Jiangxi Agricultural University, Nanchang 330045, China; 2 Institute of Kiwifruit, Jiangxi Agricultural University, Nanchang 330045, China; 3 Fruit Science Institute, College of Agriculture and Biotechnology, Zhejiang University, Hangzhou 310058, China

**Keywords:** kiwifruit, transcriptomic, metabolomics, phenolic acid, flavonoids

## Abstract

*Actinidia eriantha* is a unique germplasm resource for kiwifruit breeding. Genetic diversity and nutrient content need to be evaluated prior to breeding. In this study, we looked at the metabolites of three elite *A. eriantha* varieties (MM-11, MM-13 and MM-16) selected from natural individuals by using a UPLC-MS/MS-based metabolomics approach and transcriptome, with a total of 417 metabolites identified. The biosynthesis and metabolism of phenolic acid, flavonoids, sugars, organic acid and AsA in *A. eriantha* fruit were further analyzed. The phenolic compounds accounted for 32.37% of the total metabolites, including 48 phenolic acids, 60 flavonoids, 7 tannins and 20 lignans and coumarins. Correlation analysis of metabolites and transcripts showed *PAL* (*DTZ79_15g06470*), *4CL* (*DTZ79_26g05660* and D*TZ79_29g0271*), *CAD* (*DTZ79_06g11810*), *COMT* (*DTZ79_14g02670*) and *FLS* (*DTZ79_23g14660*) correlated with polyphenols. There are twenty-three metabolites belonging to sugars, the majority being sucrose, glucose arabinose and melibiose. The starch biosynthesis-related genes (*AeglgC*, *AeglgA* and *AeGEB1*) were expressed at lower levels compared with metabolism-related genes (*Ae*a*myA* and *AeamyB*) in three mature fruits of three varieties, indicating that starch was converted to soluble sugar during fruit maturation, and the expression level of *SUS* (*DTZ79_23g00730*) and *TPS* (*DTZ79_18g05470*) was correlated with trehalose 6-phosphate. The main organic acids in *A. eriantha* fruit are citric acid, quinic acid, succinic acid and D-xylonic acid. Correlation analysis of metabolites and transcripts showed *ACO* (*DTZ79_17g07470*) was highly correlated with citric acid, *CS* (*DTZ79_17g00890*) with oxaloacetic acid, and *MDH1* (*DTZ79_23g14440*) with malic acid. Based on the gene expression, the metabolism of AsA acid was primarily through the L-galactose pathway, and the expression level of *GMP* (*DTZ79_24g08440*) and *MDHAR* (*DTZ79_27g01630*) highly correlated with L-Ascorbic acid. Our study provides additional evidence for the correlation between the genes and metabolites involved in phenolic acid, flavonoids, sugars, organic acid and AsA synthesis and will help to accelerate the kiwifruit molecular breeding approaches.

## 1. Introduction

*Actinidia eriantha*, commonly known as kiwifruit, belongs to the genus *Actinidia*. It is a unique wild germplasm resource in China and is widely distributed in the south [1]. The fruit of *A. eriantha* is easily peeled and has a high nutrient content, e.g., the content of ascorbic acid (AsA) is 1176 mg/100 g FW, which is three or four times that in *A. chinensis* (298 mg/100 g FW) [2,3], and the content of the total phenol (1200 mg/100 g FW) is more than ten times than that in *A. chinensis* (214 mg/100 g FW) [4]. The flower of *A. eriantha* is beautiful, with the petals in different shades of pink, while the root has antitumor pharmacological properties [5,6]. It has always been recognized as a novel berry with great development potential as with *A. chinensis* and *A. deliciosa* and has good prospects for improvement of commercial production. There are abundant germplasm resources of *A. eriantha* in China, and more than 300 accessions have been recorded and evaluated [7,8]. Those resources can be used for the breeding of new *A. eriantha* cultivars and novel types of kiwifruit through inter- and intra-specific hybridization [9]. However, less than ten excellent cultivars selected from the natural individuals were reported [10], and only one hybridization cultivar, ‘Jinyan’, has been registered [11]. As the average fruit weight is only 40–50 g, and its taste is sour and slightly astringent, the commercial cultivation of *A. eriantha* is limited, so high sugar and high polyphenol contents are important breeding targets for modern *A. eriantha* breeding. We selected several elite accessions from the natural group, such as the ‘MM-11’ with high sugar content and ‘MM-13’ and ‘MM-16’ with high polyphenol content. The nutrient composition and content are important quality characteristics for commercialization and provide an index for the evaluation of fruit quality. Understanding the nutrient composition is essential for conventional breeding. Previously, studies on the evaluation of nutrient components of *A. eriantha* were mainly investigated using simple measuring methods such as HPLC (High performance Liquid Chromatography) [2,3,4,12]. In this study, the nutrient components of three *A. eriantha* lines were investigated using UPLC-MS/MS methods, which have been used for nutrient evaluation in *A. chinensis* kiwifruit [13].

Polyphenols, secondary metabolites of plants, can be divided into different classes, including phenolic acids, flavonoids, tannins, stilbenes, lignans and coumarins [14,15]. These compounds have roles in response to abiotic stress such as extreme temperatures, drought, high salt and flooding [16], and are also beneficial to human health: phenolic acids have anti-cancer, antioxidant, anti-inflammatory, anti-bacterial and antitumor properties [17,18,19], and flavonoids and lignin are related to the prevention of cardiovascular disease and cancer [20,21]. Previous studies have shown phenolic acids and flavonoids are important constituents in *A. chinensis*, *A. argute* and A. *deliciosa*, forming the material basis for the remarkable antioxidant activity of the genus [22,23,24].

**Figure 1:**
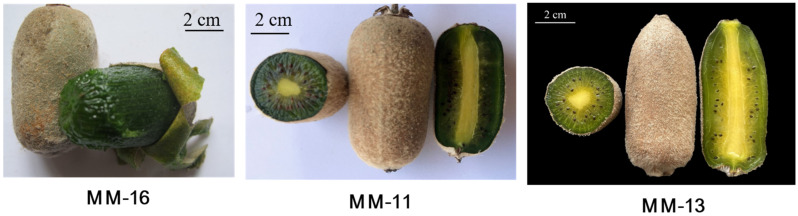
Fruit phenotypes of ‘MM-11’, ‘MM-13’ and ‘MM-16’.

Soluble sugar and organic acid are important components of kiwifruit flavor and nutritional value. Starch is typically accumulated during fruit development and degraded into soluble sugar after fruit maturity [25,26]. Multiple enzymes are involved in starch degradation, and the key enzymes vary greatly in different species, organs and development stages [27,28,29,30]. The activity changes in AdAMY1 and AdBAM3L have been shown to correlate with starch degradation in kiwifruit [26,31]. The predominant soluble sugars in the fruit also vary among species. Glucose and fructose are the main soluble sugars in *A. deliciosa*, *A. rufa* and *A. eriantha* fruits, while sucrose is the primary sugar in *A. argute* [10,32]. Previous studies have addressed the dynamic changes in starch and soluble sugar levels and gene expression that occur during kiwifruit development and ripening stages, with SPS, SPP, AGPases related to the synthesis of sucrose and starch, and SUS, FK, and HK related to metabolism reported [12,13,33]. The organoleptic properties of the fruit are determined by the type and content of soluble sugar and organic acid. The primary organic acids in kiwifruit are citric, quinic and malic acids [32]. The high content of AsA makes kiwifruit the “king of vitamin C fruit”, and its accumulation is mainly regulated by biosynthesis, cycling and degradation in kiwifruit. There are four synthetic pathways of AsA in kiwifruit, with L-galactose as the main pathway [34]. Little is known about the nutrients of *A. eriantha*. Here we use metabolome and transcriptome analyses in three varieties to expand the understanding of the nutrient metabolism mechanism in the fruit of *A. eriantha* (Figure 1).

## 2. Results

### 2.1. The Main Nutritional Components of A. eriantha Fruit Detected by Metabolomic Analyses

**Figure 2:**
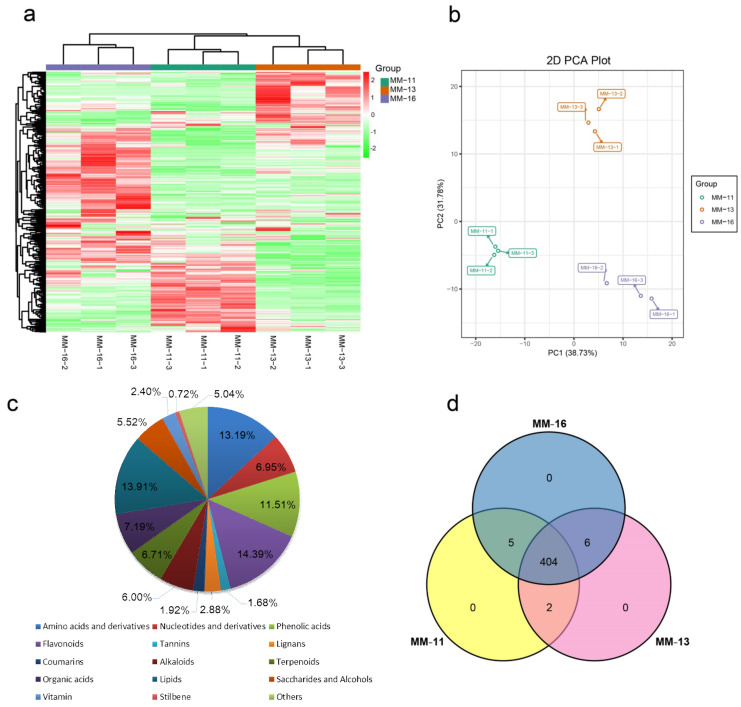
Qualitative and quantitative analysis of the metabolomics data of three varieties of fruits. (**a**) Heatmap of the quantified identified metabolites. The color scale indicates the level of metabolite accumulation. (**b**) PCA analysis of the three fruit varieties. (**c**) Component analysis of the identified metabolites from three varieties. (**d**) The Venn diagram shows the number of metabolites identified in ‘MM-11’, ‘MM-13’ and ‘MM-16’.

To better understand the metabolic profiling of *A. eriantha*, the primary and second metabolites were identified in the fruit of ‘MM-11’, ‘MM-13’ and ‘MM-16’ (Figure 1) by UPLC-MS/MS analysis (Table S1). Based on the cluster and PCA analysis, nine samples were clearly divided into three groups (Figure 2a,b). The value of PC1 and PC2 were 38.73% and 31.78%, respectively, and the two principal components accounted for 70.51% of the total variance. This suggested a difference in metabolic phenotypes among the three *A. eriantha* varieties. The large-scale analysis identified a total of 417 metabolites in MM-11, MM-13 and MM-16 samples (Table S1), which were categorized into more than 15 classes, mainly including flavonoids (14.38%), lipids (13.90%), amino acids and derivatives (13.18%), phenolic acids (11.51%), organic acids (7.19%), and terpenoids (6.71%) (Figure 2c). Phenolics, including 48 phenolic acids, 60 flavonoids, 7 tannins and 20 lignans and coumarins, accounted for 32.37% of the total metabolites. The flavonoids were composed of 25 flavonols, 18 flavonoids, 7 dihydroflavones, 5 flavanols, 3 anthocyanins, and 1 chalcone and flavonoid carbonoxide. Though 48 metabolites were phenolic acids, the main phenolic acids in *A. eriantha* were identified as vanillic acid-glucoside, quillaic acid, coniferin, protocatechuic acid-4-glucoside, 2,5-dihydroxy benzoic acid o-hexside and 3,4,5-trimethoxyphenyl-β-D-glucopyranoside. Epigallocatechin gallate and six proanthocyanidins (A3, B1, B2, B3, B4 and C2) belong to tannins (Table S1). The organic acids include 30 metabolites, and the main acids are citric acid, quinic acid, succinic acid and D-xylonic acid. Twenty-three metabolites were detected as sugars, with the main metabolite being glucose, sucrose, arabinose and melibiose (Table S1). Of the metabolites, 411 were detected in MM-11, 415 in MM-13 and 412 metabolites in MM-16 (Figure 2d). These results suggested that these three *A. eriantha* fruit varieties shared similar metabolite components but distinct metabolite expression patterns.

### 2.2. Dynamic Metabolic Changes of Different Varieties

**Figure 3:**
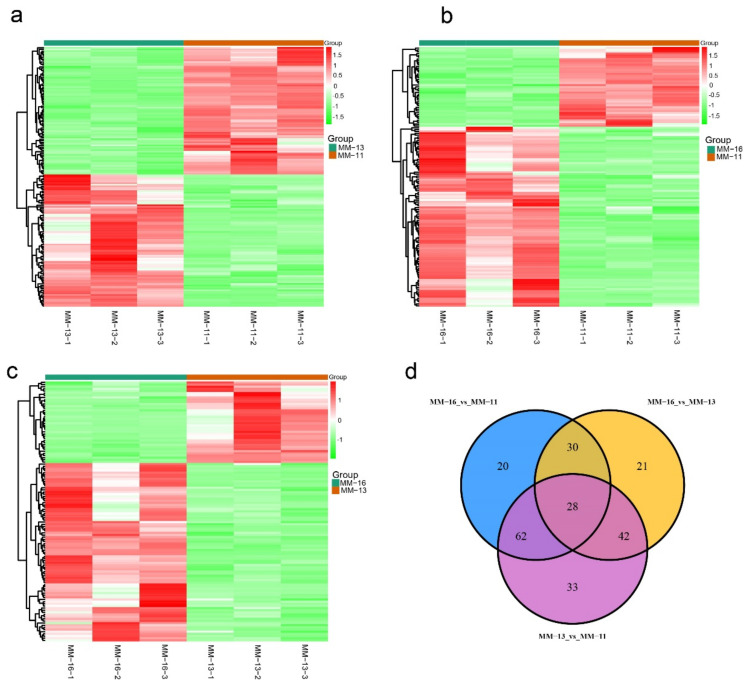
Differentially accumulated metabolites (DAMs) between ‘MM-11’, ‘MM-13’ and ‘MM-16’. Heatmap of the DAMs in MM-11 vs. MM-13, MM-11 vs. MM-16 and MM-13 vs. MM-16 are shown in (**a**–**c**), respectively. (**d**) Venn diagram showing the number of DAMs in MM-11 vs. MM-13, MM-11 vs. MM-16 and MM-13 vs. MM-16.

In order to discover the changes in the pattern of metabolite expression in the three varieties, we analyzed their DAMs with the following parameters of VIP ≥ 1.0 and fold change ≥2 or ≤0.5. As a result, a total of 165 DAMs were identified between MM-11 and MM-13, including 81 up-regulated metabolites and 84 down-regulated metabolites in MM-13 (Figure 3a and Table S2). A total of 140 DAMs were detected between MM-11 and MM-16, divided into 43 up-regulated metabolites and 97 down-regulated (Figure 3b and Table S3). Among 121 DAMs detected between MM-13 and MM-16, 38 metabolites were up-regulated and 83 down-regulated in MM-16 (Figure 3c and Table S4).

In all, 236 DAMs were identified in three comparable groups, which could be categorized into more than 15 classes. The flavonoids and phenolic acids were the majority of DAMs in the three groups (Table 1). With multiple comparative analysis, 28 DAMs were detected in the three cultivars (Figure 3d and Table S5), with almost half of them being flavonoids and phenolic acid. For the flavonoids, there were significant decreases in the content of cyanidin-3-O-rutinoside and eriodictyol C-hexoside in MM-13. The contents of narirutin, nobiletin, poncirin and isosinensetin were lower in MM-11 than MM-13 and MM-16, and chrysoeriol-O-acetylhexoside was only detected in MM-13 and MM-16. For phenolic acids, MM-11 had lower content of protocatechuic acid-4-glucoside, caffeic acid and oxalic acid compared with MM-13 and MM-16 (Table S5).

### 2.3. Transcriptome Analysis

**Figure 4:**
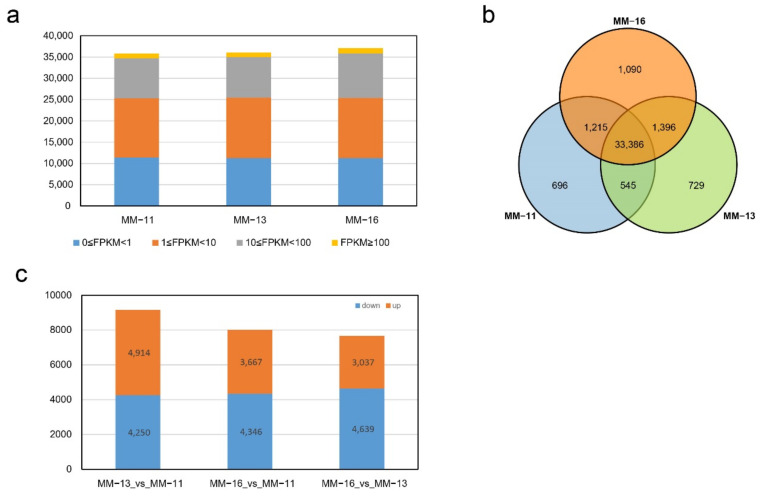
F Overview of kiwifruit MM-11, MM-13 and MM-16 fruit transcriptomes. (**a**) Distribution of genes with high expression (FPKM ≥ 100), middle expression (100 > FPKM ≥ 10), low expression (10 > FPKM ≥ 1), and no expression (1 > FPKM ≥ 0). (**b**) Venn diagram of genes expressed in MM-11, MM-13 and MM-16 samples. (**c**), Distribution of up- and down-regulated DEGs in the pair-wise MM-13 vs. MM-11, MM-16 vs. MM-11 and MM-16 vs. MM-11 analyses.

The cDNA libraries of MM-11, MM-13 and MM-16 mature fruits were constructed with three biological replicates. The 388.02 million clean reads obtained (Table S6) were aligned to a reference genome and the expression levels of the genes were quantified. About 84% of reads were successfully mapped to the reference genome, and approximately 80% of reads were uniquely mapped. The total of 39,057 expressed genes identified in MM-11, MM-13 and MM-16 all shared similar distributions of the number of genes at different expression levels (Figure 4a). Nearly 30% of these 39,057 expressed genes were not expressed in all three varieties, with the FPKM values lower than one. Genes expressed at a low level (10 > FPKM ≥ 1) accounted for the highest proportion, followed by those expressed at 100 > FPKM ≥ 10, with the highly expressed genes (FPKM ≥ 100) accounting for the lowest proportion. We compared the expressed genes in three varieties and found 85.5% were shared in MM-11, MM-13 and MM-16, while 696, 729 and 1090 genes were specifically expressed in MM-11, MM-13 and MM-16, respectively (Figure 4b). Differentially expressed genes (DEGs) among varieties were defined with |log2(fold change)| ≥1 and FDR < 0.05. A total of 14,354 DEGs were identified by two-by-two comparison of the three samples, including 9164 DEGs in MM-13 vs. MM-11, 8013 DEGs in MM-16 vs. MM-11, 7676 DEGs in MM-16 vs. MM-11 (Figure 4c).

### 2.4. Identified Metabolites Involved in Phenolic Acid, Flavonoid and Anthocyanin Biosynthesis

**Figure 5:**
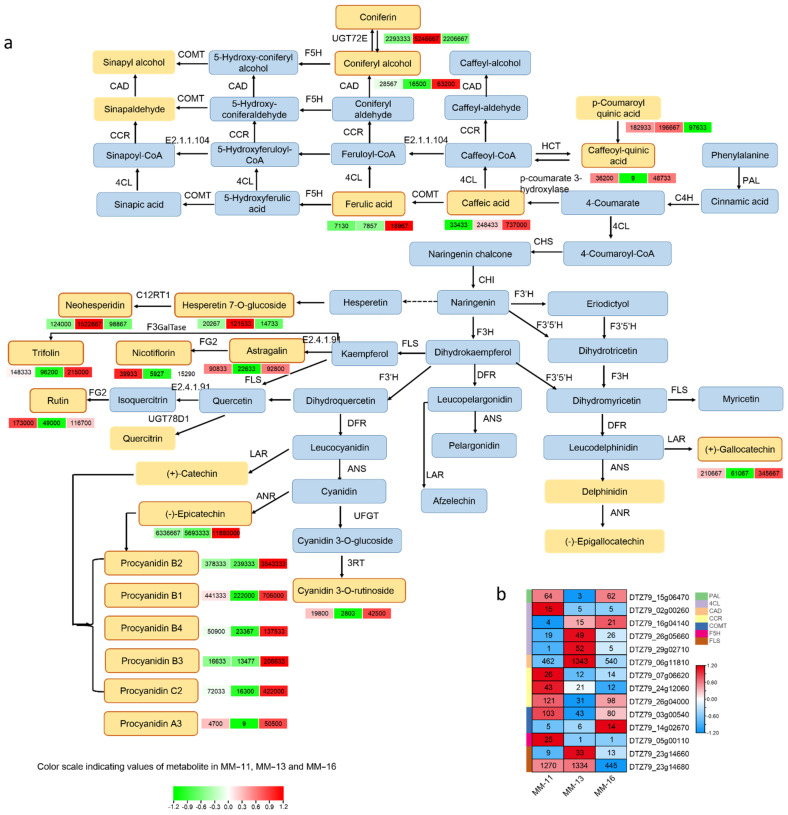
The phenolic acid, flavonoid and anthocyanin synthesis pathway of *A. eriantha* fruit. Detected metabolites are in yellow, and red squares indicate DAMs. The heatmap of DAMs (**a**) content and DEGs (**b**) in MM-11, MM-13 and MM-16 is shown. PAL, phenylalanine ammonia-lyase; C4H, cinnamic acid 4-hydroxylase; 4CL, 4-coumarate CoA ligase; CHS, Chalcone synthase; CHI, Chalcone isomerase; F3H, Flavonoid 3-hydroxylase; F3′H, Flavonoid 3′-monooxygenase; F3′5′H, Flavonoid 3′,5′-hydroxylase; DFR, Dihydroflavonol 4-reductase; ANS, Anthocyanidin synthase; ANR, anthocyanidin reductase; LAR, leucoanthocyanidin reductase; UGT78D1, flavonol-3-O-rhamnosyltransferase; FG2, flavonol-3-O-glucoside L-rhamnosyltransferase; E2.4.1.91, flavonol 3-O-glucosyltransferase; F3GalTase, kaempferol 3-O-galactosyltransferase; C12RT1, flavanone 7-O-glucoside 2′′-O-beta-L-rhamnosyltransferase; COMT, caffeate 3-O-methyltransferase; CCR, cinnamoyl-CoA reductase; CAD, cinnamyl-alcohol dehydrogenase; UGT72E, coniferyl-alcohol glucosyltransferase; HCT, shikimate O-hydroxycinnamoyltransferase; F5H, ferulate 5-hydroxylase; E2.1.1.104, caffeoyl-CoA O-methyltransferase.

A total of 28 metabolites involved in the phenolic acid, flavonoid and anthocyanin biosynthetic pathways were detected, including 8 phenolic acid, 13 flavonoids and 6 proanthocyanidins (Figure 5a and Table 2). Six of the twenty-one DAMs detected belong to the phenolic acid pathway (coniferin, caffeic acid, coumaroyl quinic acid, sinapyl alcohol, coniferyl alcohol, sinapinaldehyde, ferulic acid and caffeoylquinic acid), nine DAMs belong to flavonoids and six to proanthocyanidins. Among the nine flavonoids, one belongs to Dihydroflavone (neohesperidin), one to flavonoid (hesperetin-7-O-glucoside), two belong to anthocyanins (delphinidin, cyanidin-3-O-rutinoside), four to flavanols (epicatechin, catechin, gallocatechin and epigallocatechin), and five to flavonols (trifolin, rutin, quercitrin, astragalin, nicotiflorin). The six proanthocyanidins include procyanidin A3, B1, B2, B3, B4 and C2. All eight metabolite contents of phenolic acid were highest in MM-13, while 13 metabolite contents of flavonoids and 6 of proanthocyanidins were highest in MM-16 (Table 2).

### 2.5. Expression of Phenolic Acid, Flavonoid and Aanthocyanidin Biosynthesis Genes in Three A. eriantha Varieties

We searched phenolic acid, flavonoid and anthocyanin biosynthesis pathways of kiwifruits based on the detected metabolites in reference to the phenylpropanoid biosynthesis pathway in the KEGG database (Figure 5). A total of 80 genes were found to be involved in these pathways (Figure S1a). Comparison of the 64 DEGs identified in the phenolic acid, flavonoid and anthocyanidin biosynthesis pathways revealed the difference of phenolics in the three varieties (Figure S1b). Among them, 47 were in MM-11 vs. MM-13, 39 DEGs in MM-11 vs. MM-16 and 43 DEGs were in MM-13 vs. MM-16. High FPKM and high fold change enhance the flux in the phenylpropanoid, flavonoid and anthocyanidin biosynthetic pathways. The expression of the structural gene *PAL* (*DTZ79_15g06470*) was higher in MM-16 and MM-11 than MM-13, and with a 19.35- and 18.75-fold upregulation in MM-11 vs. MM-13 and MM-16 vs. MM-13, respectively. *FLS* (*DTZ79_23g14680*) was more highly expressed in MM-11 and MM-13 than MM-16. The expression level of *CAD* (*DTZ79_06g11810*) was high in MM-13, which supports the high accumulation of coniferin in MM-13. From the correlation analysis between transcriptome and metabolite data, a total of nine candidate DEGs were found highly correlated with the corresponding metabolites, including *PAL* (*DTZ79_15g06470*), 4CL (*DTZ79_26g05660* and *DTZ79_29g0271*), CAD (*DTZ79_06g11810*), COMT (*DTZ79_14g02670*) and *FLS* (*DTZ79_23g14660*) (Figure 5b and Table 3).

**Figure 6:**
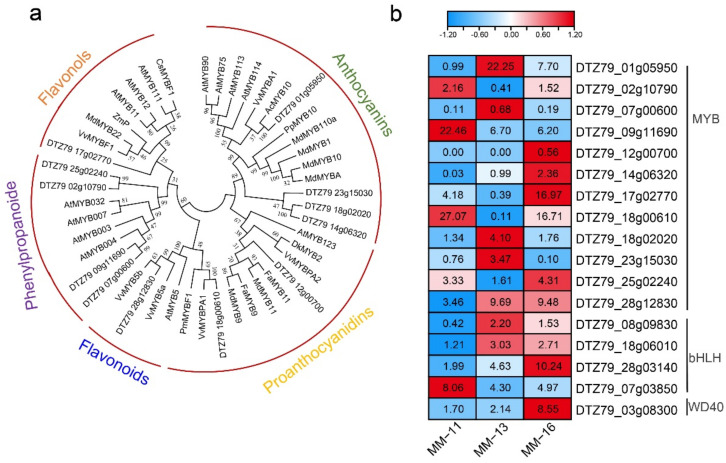
Transcription factors involved in phenolic acid, flavonoid and anthocyanin biosynthesis. (**a**) Phylogeny of MYBs in the anthocyanin, proanthocyanidin, phenylpropanoid, flavonols and flavonoids clades. (**b**) Heatmap of the MYB, bHLH and WD40 transcription factors genes.

MYB, bHLH and WD40 transcription factor families play roles in regulating the expression of the structural genes in phenylpropanoid, flavonoid and anthocyanins biosynthesis pathways [35,36]. The MYB and bHLH genes of *A. eriantha* were selected to construct a phylogenetic tree with Arabidopsis MYB TFs and function bHLH TFs in other species, respectively. We identified 12 MYBs involved in phenylpropanoid, flavonoid and anthocyanins biosynthesis pathways (Figure S2). Phylogenetic analysis of functional MYBs from other species and 12 *AeMYB*s showed they can be divided into five clusters (Figure 6a), and their expression level in the three varieties is shown in Figure 6b. *DTZ79_01g05950* had homology with *AcMYB10* (*Acc00493*) and about 22-fold and 7.7-fold higher expression in MM-13 and MM-16 than in MM-11. Three bHLH TFs were clustered in the functional bHLH clade (Figure S3). *DTZ79_28g03140* (*bHLH*) encodes a protein that is highly homologous to the protein of *AcbHLH5* (*Acc19563*), and *DTZ79_03g08300* encodes a protein that is highly homologous to VvWDR1. We also identified one WD40 involved in phenylpropanoid, flavonoid and anthocyanins biosynthesis pathways (Figure 6b). The expression of four MYB genes (*DTZ79_07g00600*, *DTZ79_12g00700*, *DTZ79_02g10790* and *TZ79_18g00610*) and *WD40* (*DTZ79_03g08300*) were highly correlated with structural genes (Table 3).

### 2.6. Soluble Sugar, Organic Acid and AsA in Three A. eriantha Varieties

**Figure 7:**
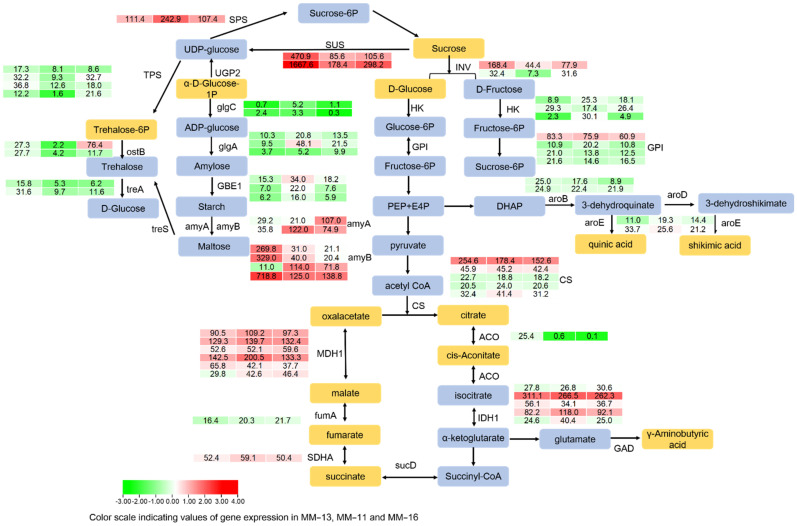
F Transcript profiling of genes in the starch, sucrose and organic acid biosynthetic pathways in MM-11, MM-13 and MM-16 at mature stages. The heatmap of DEGs was analyzed, and FPKM values of genes are shown.

Soluble sugar and organic acid are the main determinants of fruit taste and also important regulatory signals of fruit ripening. During the fruit maturation stage, starch begins to hydrolyze and sugars accumulate in kiwifruit [13]. The sugar and organic acid metabolism pathways of *A. eriantha* fruits were constructed based on the detected metabolites in reference to the starch, sucrose and organic acid biosynthesis in the KEGG database (Figure 7). Thirteen metabolites involved in the starch, sucrose and organic acid biosynthesis were detected, including nine organic acids (citric acid, quinic acid, succinic acid, γ-aminobutyric acid, malic acid, trans-citridic acid, fumaric acid, shikimic acid and oxaloacetic acid) and four saccharides (glucose, sucrose, glucose-1-P and trehalose 6-P) (Figure 7). Four DAMs found by the two-by-two comparison of the three varieties were succinic acid, malic acid, trans-citridic acid and shikimic acid. The expression of starch biosynthesis-related genes, including *AeglgC*, *AeglgA* and *AeGEB1*, were at low or mid-levels (FPKM < 100) in the three varieties (Figure 7). However, the expression of *AeamyA* and *AeamyB,* which hydrolyze starch into maltose, was high. We identified seven *AeamyB* and two *AeamyA* genes, with *AeamyB* generally more highly expressed than *AeamyA.* The expression of *DTZ79_24g11560* (*amyB*) was consistent with the content of rehalose 6-phosphate (Table 3). The expression of sucrose synthase (*SUS*) and sucrose phosphate synthase (*SPS*), the key enzymes in sucrose biosynthesis, was high, with the FPKM value of more than 100. Both sucrose synthase genes were more highly expressed in MM-11 than in MM-13 and MM-16. Invertase (Inv) can hydrolyze sucrose into glucose and fructose, and hexokinase (HK) catalyzes fructose and glucose [37]. The expression of *AeInv* was higher in MM-11 and MM-16 compared to MM-13, with a mid-level of expression, while the expression of *AeHK* was lower than that of *AeInv*. These results suggested that starch was converted to soluble sugar during fruit maturation.

The main organic acid in the *A. eriantha* fruit included quinic acid, citric acid, succinic acid, malic acid and γ-Aminobutyric acid. We scanned the expression of genes related to organic acid synthesis. *AeCS*, *AeMDH1* and *AeIDH1* were more highly expressed in all three, while expression of the quinic acid and shikimic acid-related genes aroB and aroD were at a middle level (Figure 7).

**Figure 8:**
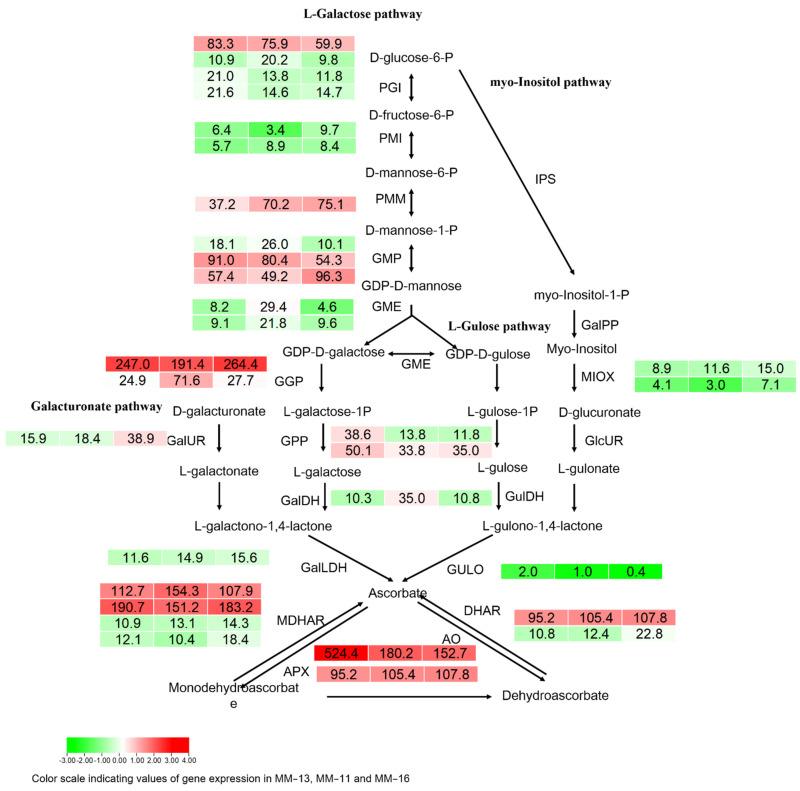
Ascorbic acid biosynthesis pathways and recycling pathways in kiwifruit. The heatmap of DEGs was analyzed, with FPKM values of genes in boxes.

As kiwifruit is one of the highest nutritional value fruits as it is rich in ascorbic acid (vitamin C, AsA), we investigated the genes involved in the four ascorbic acid biosynthesis pathways and recycling pathways in kiwifruit: the L-galactose pathway, D-galacturonic acid pathway, inositol pathway and L-gulose pathway (Figure 8) [38]. The *AeGGP* (GDP-L-galactose phosphorylase) gene had a high expression level, as did the *AeAO*, *AeAPX*, *AeMDHAR* and *DHAR* genes involved in the ascorbic acid recycling pathway.

### 2.7. Validation of Gene Expression Level in Three Varieties

To confirm the gene expression pattern of the *A. eriantha* fruit identified by RNA-Seq data, ten DEGs were randomly selected for validation using qRT-PCR, with the primers shown in Table S7. The results of the expression pattern of qPCR were consistent with the RNAseq data (Figure S4), which confirmed that the gene expression profile of RNA-seq data was reliable.

## 3. Discussion

Metabolome and transcriptome analyses were widely used for studies of sugar, flavor, flavonoids and anthocyanin formation and early maturation traits in kiwifruit [13,39,40,41]. Here, we firstly investigated the nutritional components of *A. eriantha* based on metabolome and transcriptome sequencing, then comprehensively analyzed the main nutrient component of *A. eriantha* fruit, including phenolic acid, flavonoids, sugars, organic acid and AsA acid. A total of 417 metabolites were identified, and 135 of them were found to be phenolic compounds. These compounds are strong antioxidants that have been found in kiwifruit, with the content higher in *A. eriantha* than in *A. deliciosa* and *A. chinensis* [4,22]. Phenolic acids and flavonoids are the major secondary metabolites in fruits [14,15,42,43], and the biosynthesis of phenolic compounds in plants is primarily derived via the phenylpropanoid pathway [44]. PAL, C4H and 4CL are major enzymes involved in the biosynthesis of many secondary metabolites, including phenolic acid, flavonoids and lignin. F5H, COMT, CAD, CCR and UGT72E enzymes mediate the synthesis of different phenolic acid derivatives. CHS, CHI, F3H, FLS, DFR and ANS enzymes are involved in flavonoids [45]. LAR and ANR enzymes lead to catechin and epicatechin, respectively [46,47]. In our study, *PAL* (*DTZ79_15g06470*), 4CL (*DTZ79_26g05660* and *DTZ79_29g0271*), CAD (*DTZ79_06g11810*), COMT (*DTZ79_14g02670*) and *FLS* (*DTZ79_23g14660*) were highly correlated with the corresponding metabolites, suggesting they may play roles in regulating the synthesis of phenolic compounds in *A. eriantha*. Previous studies have shown that transcription factors, including MYB, bHLH and WD40, regulate the phenylpropanoid, flavonoid and anthocyanin biosynthesis pathways in different plants [44,48]. The homologs of *DTZ79_28g03140* and *DTZ79_01g05950* in *A. chinensis* were *AcbHLH5* (*Acc19563*) and *AcMYB10* (*Acc00493*), respectively. *AcbHLH5* has been reported to be the partner protein of AcMYB10 and AcMYB110 and plays roles in the regulation of anthocyanin accumulation [41,49,50,51]. *AchMYC2*, the homolog of *DTZ79_07g03850*, can activate *PAL*, *C4H* and 4*CL* expression in kiwifruit [52]. *DTZ79_18g00610* is close to *VvMYBPA1*, which has been reported to regulate proanthocyanidin synthesis during fruit development in grape [53,54]. *DTZ79_28g03140*, *DTZ79_01g05950*, *DTZ79_07g03850* and *DTZ79_18g00610* may also play roles in the regulating structural genes in flavonoid biosynthesis in *A. eriantha*.

Based on the metabolic characteristics of kiwifruit, starch is accumulated in the development stage, while in the maturation and ripening stages, starch is degraded and glucose, fructose and sucrose are accumulated [26,55]. In our study, the expression of the starch synthesis-related genes *glgC*, *glgA*, *GBE1* was low compared with the starch degradation-related genes *amyA* and *amyB*, and the sucrose synthesis-related genes *SPS* and *SUS* were highly expressed (Figure 5). These results are consistent with the conversion of starch into sugar during the maturation and ripening stage of kiwifruit. The homolog of the *amyB* genes *DTZ79_14g07050* and *DTZ79_14g07230* in *A. deliciosa* is *AdBAM3L,* which is a key structural gene in starch degradation [26,31]. These two genes may also have roles in starch degradation, and this needs to be further investigated. The organic acid types and component contents of kiwifruit vary among genotypes [56]. In the ripening stage, the main organic acid accumulated in MM-11, MM-13 and MM-16 fruits included quinic acid, citric acid and malic acid, as with other *Actinidia* species [32]. The *MDH1* (malate dehydrogenase) and IDH1 (isocitrate dehydrogenase) had a high expression level, which may be consistent with the high content of citric acid and malic acid in mature fruit. The expression of *DTZ79_23g14440* (*MDH1*) was highly correlated with malic acid (r = 0.99), which may be the key structural gene in regulation of malic acid biosynthesis. Kiwifruit have an extremely high content of ascorbic acid, which varies among genotypes [57]. Generally, the content of AsA acid is higher in *A. eriantha* fruit than in *A. chinensis* or *A. deliciosa* [58]. In the present study, we investigated genes in the AsA biosynthesis and recycling pathways (Figure 8). The *AeGGP* (GDP-L-galactose phosphorylase) genes were expressed two to eight times more than the other genes in the AsA biosynthesis pathway, indicating the L-galactose pathway is the major route of AsA synthesis in kiwifruit [58]. The expression of the genes involved in the L-galactose pathway was higher than that in the other three pathways. We also detected genes involved in the AsA recycling pathway, including *MDHAR* (monohydroascorbate reductase) and *DHAR* (droascorbate reductase), which are responsible for ascorbic acid regeneration from its oxidized forms, and *APX* (L-ascorbate peroxidase) and *AO* (L-ascorbate oxidase) that oxidize AsA to monohydroascorbate and dehydroascorbate, respectively. Our study provides a comprehensive insight into the main nutrient component of *A. eriantha* fruit. Based on the genes highly correlated to phenolic acid, sugar, organic acid and ascorbic acid, specific molecular markers will be developed and applied to efficient and targeted kiwifruit selection, evaluation and improvement.

## 4. Materials and Methods

### 4.1. Fruit Materials

Three varieties of *A. eriantha*, ‘MM-11’, ‘MM-13’ and ‘MM-16’, were used for transcriptome and metabolomics sequencing. All three varieties were more than 3 years old after grafting on 8-year-old ‘Jinkui’ (*A. deliciosa)* rootstock in a Shanwei kiwifruit germplasm resource nursery located in Fengxin County (28° N, 114° E, elevation 76 m) Jiangxi province, PR China. Mature fruit of the three varieties were collected with three biological replicates and immediately frozen in liquid nitrogen and stored at −80 °C for RNA-Seq and metabolic assays.

### 4.2. Metabolome Data Analysis

Three replicates of ripe fruit of MM-11, MM-13 and MM-16 were used for metabolite profiling with technology of the Wuhan Metware Biotechnology Co., Ltd. (Wuhan, China) (http://www.metware.cn/) (accessed on 5 August 2022), using a widely targeted metabolome method. The freeze-dried fruit was crushed into powder using a mixer mill (MM400, Retsch) and 100 mg of powder was extracted overnight at 4 °C with 0.6 mL 70% aqueous methanol. Following centrifugation at 10,000× *g* for 10 min, the extracts were filtered and then analyzed on an UPLC-MS/MS system with the following analytical conditions: UPLC: column, Agilent SB-C18. The mobile phase consisted of pure water (Solvent A) and acetonitrile (Solvent B), both acidified with 0.1% (*v*/*v*) formic acid. The mobile phase gradient was run from 5% Solvent B at 0 min to 95% Solvent B at 9.0 min and maintained for 1 min, then the 5% Solvent B was adjusted at 10–11.1 min and maintained for 3.9 min. The flow velocity was set as 0.35 mL/min. The column oven temperature was adjusted to 40 °C, and the injection volume was 4 μL. The effluent was connected to an ESI-triple quadrupole-linear ion trap (Q TRAP)-MS, then operated in positive and negative ion mode and controlled by Analyst 1.6.3 software (AB Sciex, Concord, ON, Canada). The ESI source operation following parameters: ion source temperature was 550 °C, ion spray voltage (IS) 5500 V (positive ion mode)/−4500 V (negative ion mode). Ion source gas I (GSI), gas II (GSII) and curtain gas (CUR) were set at 50, 60 and 25 psi, respectively. The collision-activated dissociation (CAD) was high. Instrument tuning and mass calibration was performed with 10 and 100 μmol/L polypropylene glycol solution in QQQ and LIT modes, respectively. QQQ scans were using MRM models with collision gas (nitrogen) set to medium. Quantification of metabolites was carried out using an MRM method. The differentially accumulated metabolites (DAMs) were identified with the following criteria: variable importance in projection (VIP) ≥ 1 and a fold change ≥2 or ≤0.5.

### 4.3. Transcriptome Sequencing and Analysis

A total of nine samples (three replicates each of MM-11, MM-13 and MM-16) were prepared for RNA extraction based on the instructions of the Quick RNA isolation Kit (Waryong, Beijing). The quality of total RNA was analyzed using the Agilent Bioanalyzer 2100 system (Agilent Technologies, Santa Clara, CA, USA). Sequencing libraries were prepared with a NEBNext Ultra RNA Library Prep Kit for Illumina (NEB, Ipswich, MA, USA) following the manufacturer’s instructions. Transcriptome sequencing was performed on an Illumina HiSeq 2500 platform with PE125 from the Wuhan Metware Biotechnology Co., Ltd. (Wuhan, China). After removing the adapter sequences, ambiguous nucleotides, and low-quality sequences, the clean reads from each library were mapped to the *A. eriantha* (White) genome (http://kiwifruitgenome.org/) (accessed on 5 August 2022) using HISAT2 [59] to calculate the mapping ratio. The level of gene expression was measured using FPKM (fragments per kilobase per million reads) [60]. Differentially expressed genes (DEGs) between two groups were identified using the DESeq2 R package, with a minimal two-fold difference in expression (|log2 Ratio| ≥ 1) and *p*-value < 0.05. Gene annotation was performed using the NCBI non-redundant protein sequences (Nr) (https://ftp-private.ncbi.nlm.nih.gov), the protein family (Pfam) (https://pfam.xfam.org/) (accessed on 5 August 2022), the Swiss-Prot protein database (http://www.expasy.ch/sprot) (accessed on 5 August 2022)and the Kyoto Encyclopedia of Genes and Genomes (KEGG) (https://www.genome.jp/kegg/) (accessed on 5 August 2022). Gene ontology (GO) annotation was used for sequences with a match in the Nr database by using Blast2GO v.3.0 [61]. The GO enrichment and KEGG enrichment analysis of DEGs were conducted using TBtools, with FDR < 0.05 regarded as significantly enriched in DEGs [62]. Heatmaps were prepared using TBtools [62]. Correlation analysis of transcriptome and metabolite data were performed by using excel, with a Pearson correlation coefficient (PCC) > 0.9 and *p*-value <0.05. The data were log2-transformed before correlation analysis.

### 4.4. Quantitative Real-Time PCR (qRT-PCR) Validation

The total RNA of MM-11, MM-13 and MM-16 were extracted using the CTAB method. The quality and concentration of RNA were checked on a gel and measured using a BioDrop spectrophotometer (Biochrom, Cambridge, UK). The qRT-PCR primers were designed using Primer3 software (http://primer3.ut.ee/) (accessed on 5 August 2022). The qRT-PCR reaction was performed on an ABI PRISM 7900HT sequence detection system (Applied Biosystems, Waltham, MA, USA) using the SYBR Premix Ex Taq Kit (TaKaRa, Tokyo, Japan) with the following reaction conditions: 95 °C for 3 min, 40 cycles of 95 °C for 15 s, 56 °C for 30 s, and 72 °C for 20 s. Actin was used for normalization, and the expression data were calculated by the 2^∆∆Ct^ formula [63].

## 5. Conclusions

China has abundant germplasm resources of *A. eriantha*, which can be explored for modern kiwifruit breeding. Metabolomics sequencing is a good method for evaluating the nutrient profile of elite accession of *A. eriantha* prior to breeding. In the present study, we investigated the metabolites of three *A. eriantha* elite lines by using a UPLC-MS/MS-based metabolomics approach and transcriptome. We identified a total of 417 metabolites, including 135 polyphenols, 32 sugar-related metabolites, 30 organic acids. Further analysis of the biosynthesis and metabolism of phenolic acid, flavonoids, sugars, organic acid and ascorbic acid in *A. eriantha* fruit indicated that starch was converted to soluble sugar during fruit maturation, and the L-galactose pathway is the primary metabolism of ascorbic acid. Furthermore, the expression level of *PAL*, *4CL*, *CAD*, *COMT* and *FLS* genes were highly correlated with polyphenols content; *ACO*, *CS*, *fumA*, *MDH1*, and *GPI* were correlated with organic acid. *SUS* and *TPS* were responsible for trehalose 6-phosphate, and *GMP* and *MDHAR* were related to ascorbic acid. Our study provides additional evidence for the correlation between genes and traits. Molecular markers associated with traits can be further developed and used in molecular breeding approaches for kiwifruit.

## Figures and Tables

**Table 1:** Statistical analyses of differentially accumulated metabolites in MM-11, MM-13 and MM-16.

Class	MM-13 vs.MM-11)		MM-16 vs. MM-11)		MM-16 vs. MM-13)	
	Up	Down	Up	Down	Up	Down
Flavonoids	20	16	4	10	14	19
Lipids	12	7	7	2	6	1
Phenolic acids	9	13	9	18	5	14
Amino acids and derivatives	6	17	7	15	6	5
Terpenoids	6	11	5	11	1	6
Nucleotides and derivatives	6	2	2	6	0	7
Organic acids	6	3	2	7	1	7
Others	4	4	1	4	1	3
Tannins	3	0	0	4	0	6
Alkaloids	2	6	1	7	0	5
Coumarins	2	1	0	4	1	4
Saccharides and Alcohols	2	1	2	1	1	0
Vitamin	2	2	1	1	0	1
Lignans	1	1	2	7	1	5
Stilbene	0	0	0	0	1	0
Total	81	84	43	97	38	83

**Table 2:** Detected metabolites involved in phenylpropanoid, flavonoid and anthocyanins biosynthesis.

Compounds	Class	Content			Type
		MM-13	MM-11	MM-16	
Coniferin	Phenolic acids	5,246,667	2,293,333	2,206,667	DAM
Caffeic acid	Phenolic acids	248,433	33,433	737,000	DAM
3-O-p-Coumaroyl quinic acid	Phenolic acids	196,667	182,933	97,633	DAM
Sinapyl alcohol	Phenolic acids	212,333	388,000	281,000	
Coniferyl alcohol	Phenolic acids	16,500	28,567	63,200	DAM
Sinapinaldehyde	Phenolic acids	12,120	20,200	18,267	
Ferulic acid	Phenolic acids	7857	7130	18,967	DAM
4-Caffeoylquinic acid *	Phenolic acids	9	36,200	48,733	DAM
Hesperetin 7-O-neohesperidoside (Neohesperidin) *	Dihydroflavone	1,522,667	124,000	98,867	DAM
Delphinidin chloride	Anthocyanins	14,567	17,067	15,933	
Cyanidin-3-O-rutinoside (Keracyanin)	Anthocyanins	2803	19,800	42,500	DAM
Hesperetin-7-O-glucoside	Flavonoid	121,533	20,267	14,733	DAM
Kaempferol-3-O-galactoside (Trifolin)	Flavonols	96,200	148,333	215,000	DAM
Quercetin-3-O-rutinoside (Rutin)	Flavonols	49,000	173,000	116,700	DAM
Quercetin-3-O-α-L-rhamnoside (Quercitrin)	Flavonols	29,267	15,243	25,867	
Kaempferol-3-O-glucoside (Astragalin)	Flavonols	22,633	90,833	92,800	DAM
Kaempferol-3-O-rutinoside (Nicotiflorin)	Flavonols	5927	39,933	15,290	DAM
Epicatechin *	Flavanols	5,693,333	6,336,667	11,880,000	DAM
Catechin *	Flavanols	4,053,333	3,630,000	6,743,333	
Gallocatechin *	Flavanols	61,067	210,667	345,667	DAM
Epigallocatechin *	Flavanols	42,400	45,200	51,633	
Procyanidin B1	Proanthocyanidins	222,000	441,333	706,000	DAM
Procyanidin A3	Proanthocyanidins	9	4700	50,500	DAM
Procyanidin B2	Proanthocyanidins	239,333	378,333	3,543,333	DAM
Procyanidin B4	Proanthocyanidins	23,367	50,900	137,533	DAM
Procyanidin B3	Proanthocyanidins	13,477	16,633	206,633	DAM
Procyanidin C2	Proanthocyanidins	16,300	72,033	422,000	DAM

Note: *, isomer.

**Table 3:** Pearson’s correlation coefficients between gene and metabolite.

Gene Name	Gene ID	Matoblics	Correlation	*p*-Value
*4CL*	*DTZ79_29g02710*	Delphinidin chloride	−0.99905815	0.027632
*4CL*	*DTZ79_26g05660*	Quercetin-3-O-rutinoside (Rutin)	−0.99996421	0.005386
*CAD*	*DTZ79_06g11810*	Sinapinaldehyde	−0.99866962	0.032842
*COMT*	*DTZ79_14g02670*	Ferulic acid	0.999602413	0.017953
*FLS*	*DTZ79_23g14680*	Procyanidin B3	−0.99957299	0.018605
*LAR*	*DTZ79_13g00660*	Epigallocatechin	−0.99999998	0.000135
*PAL*	*DTZ79_15g06470*	4-Caffeoylquinic acid	0.99921024	0.025303
*PAL*	*DTZ79_15g06470*	Kaempferol-3-O-glucoside (Astragalin)	0.999746251	0.014342
*ACO*	*DTZ79_17g07470*	Citric Acid	−0.99901742	0.028224
*ACO*	*DTZ79_17g07470*	L-(-)-Malic acid	0.997522695	0.04482
*CS*	*DTZ79_12g12350*	L-(-)-Malic acid	0.998746142	0.031883
*CS*	*DTZ79_17g00890*	Oxaloacetic acid	−0.99920132	0.025446
*fumA*	*DTZ79_17g10730*	L-(-)-Malic acid	−0.99975265	0.01416
*MDH1*	*DTZ79_23g14440*	L-(-)-Malic acid	0.997955145	0.040719
*GPI*	*DTZ79_04g02580*	Quinic Acid	−0.99814646	0.038767
*SUS*	*DTZ79_23g00730*	Trehalose 6-phosphate	0.999359474	0.022787
*TPS*	*DTZ79_18g05470*	Trehalose 6-phosphate	0.997125182	0.048284
*GMP*	*DTZ79_24g08440*	L-Ascorbic acid	0.999898684	0.009062
*MDHAR*	*DTZ79_27g01630*	L-Ascorbic acid	0.999853612	0.010893

## Data Availability

The RNA-sequencing data have been deposited in the NCBI Sequence Read Archive. (http://www.ncbi.nlm.nih.gov/sra/) (accessed on 5 August 2022), with the accession number SAMN30608900, SAMN30608901 and SAMN30608902 for MM-11, MM-13 and MM-16, respectively.

## Supplementary Materials

The following supporting information can be downloaded at: https://www.mdpi.com/article/10.3390/ijms231810217/s1.
