# Supplementary figures and images for: Nutritional Component Analyses in Different Varieties of Actinidia eriantha Kiwifruit by Transcriptomic and Metabolomic Approaches
Source: Int J Mol Sci. 2022 Sep 6;23(18):10217. doi: 10.3390/ijms231810217 (PMC9499367; doi:10.3390/ijms231810217)

Figure S1

a

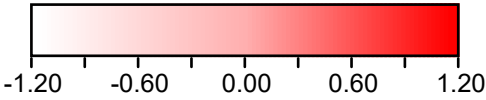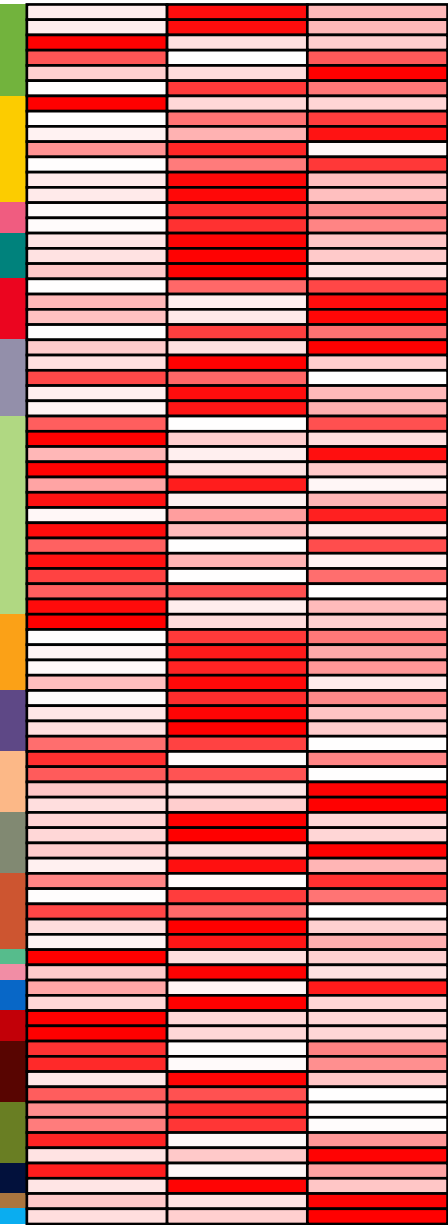

MM-11 MM-13 MM-16

b

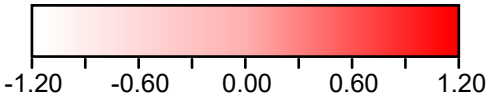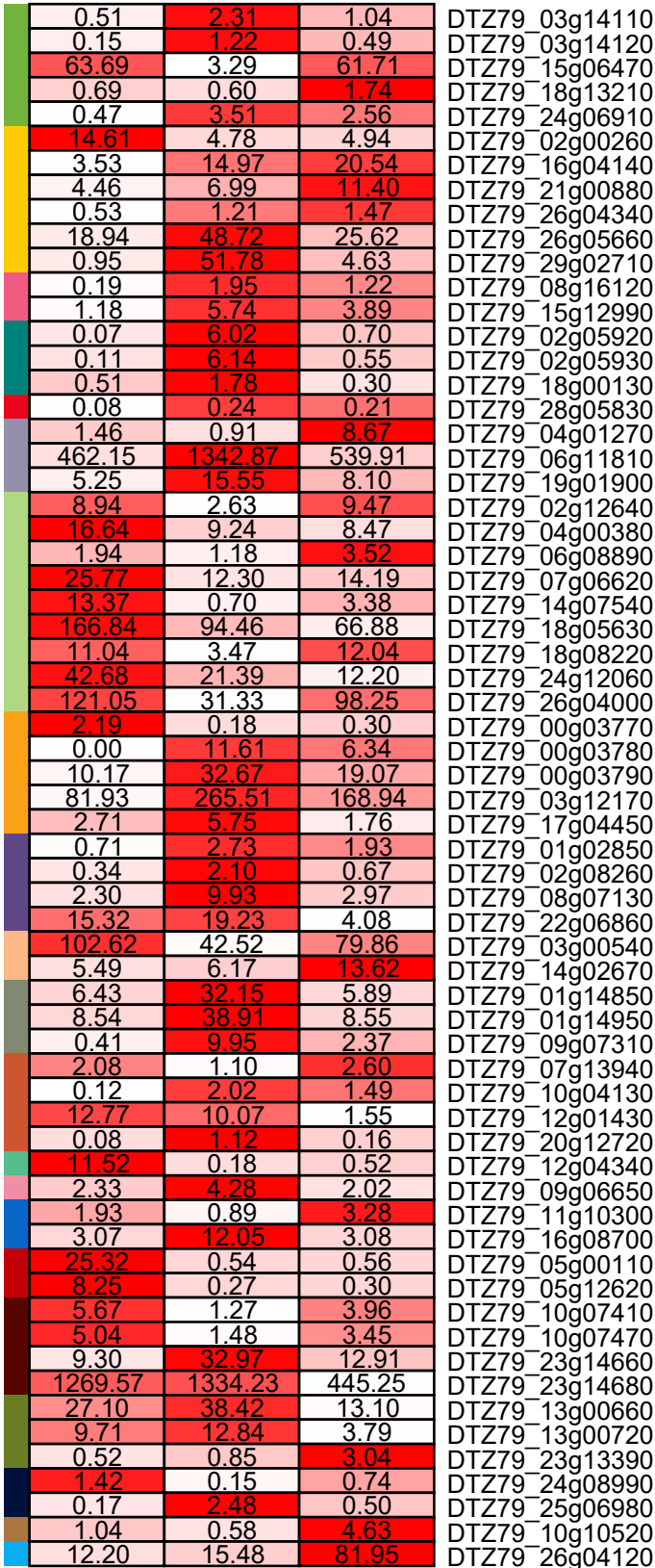

MM-11 MM-13 MM-16

Figure S2

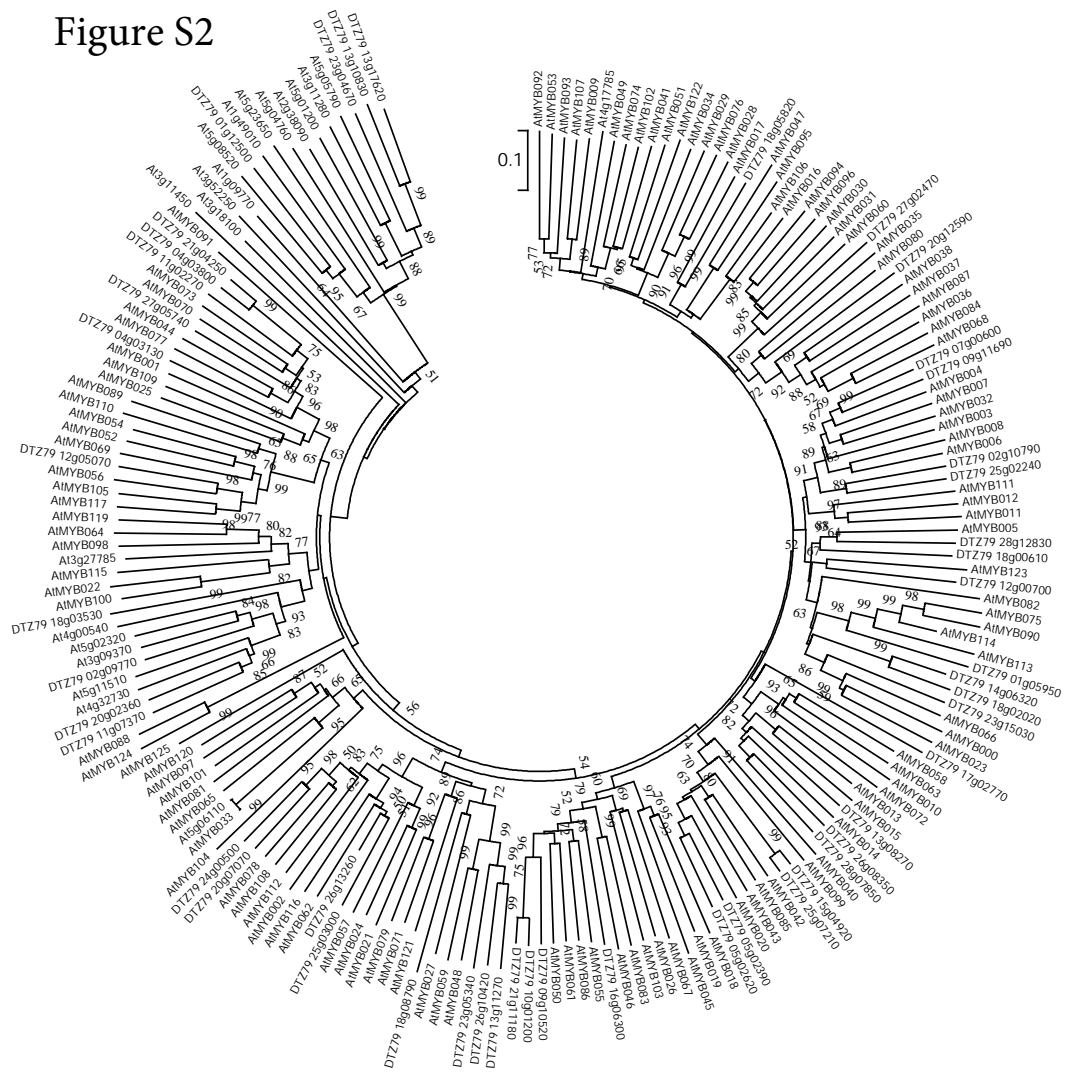



Figure S4

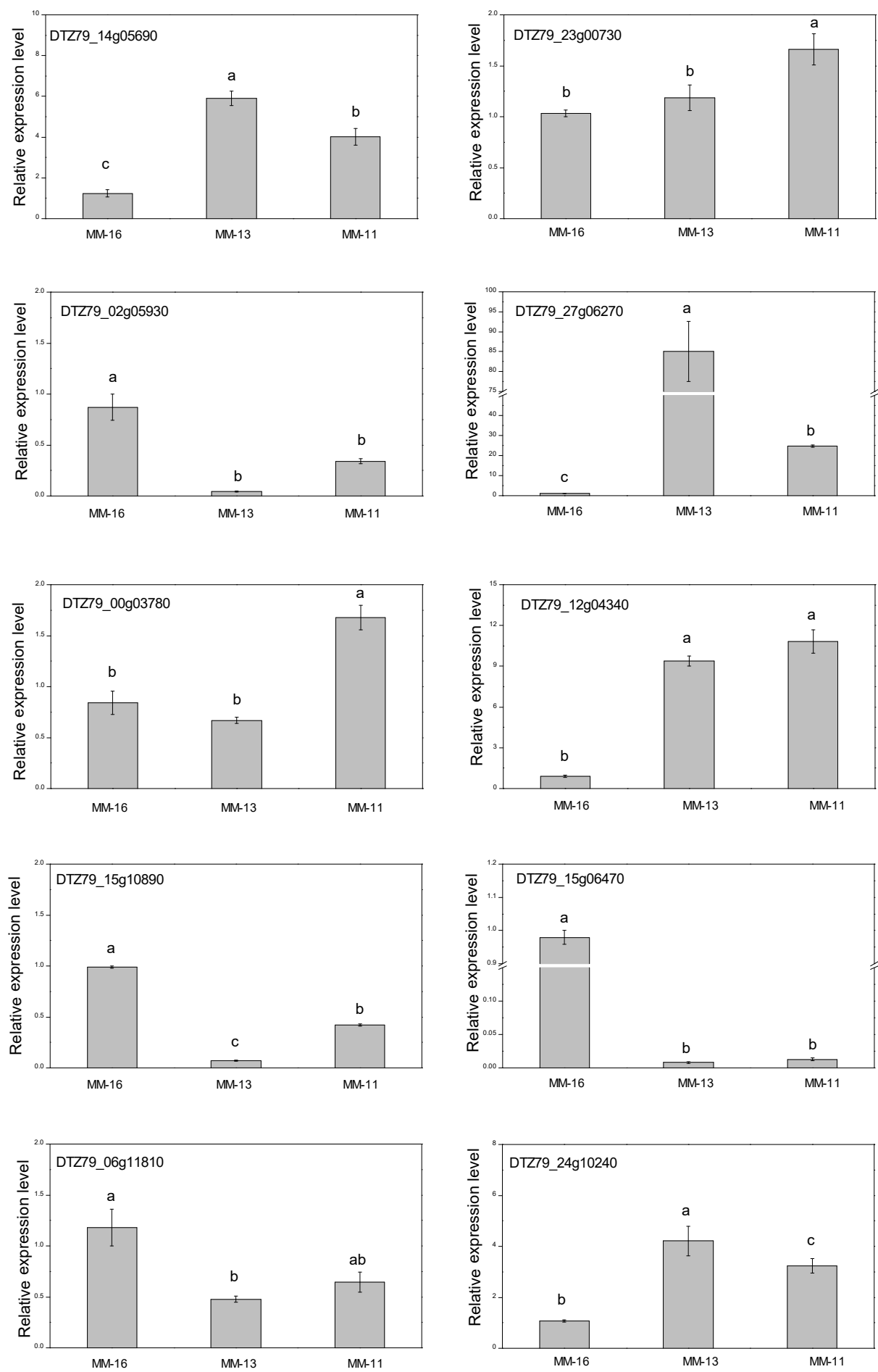

Supplement: Supplementary file 1 [file ijms-23-10217-s001.zip › All Figrue S..pdf]
